# The selective adsorption performance and mechanism of multiwall magnetic carbon nanotubes for heavy metals in wastewater

**DOI:** 10.1038/s41598-021-96465-7

**Published:** 2021-08-19

**Authors:** Zhongbing Wang, Wenbin Xu, Fanghui Jie, Zongwen Zhao, Kai Zhou, Hui Liu

**Affiliations:** 1 School of Metallurgy & Environment, Central South University, Changsha, 410083 Hunan China; 2 Dongjiang Environmental Co., Ltd., Shenzhen, 518057 Guangdong China; 3 Jiangxi Ganchang Evaluation and Testing Technology Consulting Co., Ltd., Nanchang, 330063 Jiangxi China; 4 Postdoctoral Mobile Station of Central South University, Changsha, 410083 Hunan China; 5 Shandong Humon Smelting Co. Ltd., Yantai, 264109 Shandong China

**Keywords:** Chemical engineering, Nanoscale materials, Structural materials

## Abstract

The safe treatment of heavy metals in wastewater is directly related to human health and social development. In this paper, a new type of recyclable adsorbent is synthesized through the oxidation of enhancer and modification with magnetic nanoparticles. The new adsorbent not only inherits the advantages of multiwall carbon nanotubes (6O-MWCNTs), but also exhibits a new magnetic property and further improved adsorption capacity, which is conducive to the magnetic separation and recovery of heavy metals. The adsorption results indicate that multiwall magnetic carbon nanotubes (6O-MWCNTs@Fe_3_O_4_) have a good performance for Pb(II) selective adsorption, with a maximum adsorption capacity of 215.05 mg/g, much higher than the existing adsorption capacity of the same type of adsorbents. Under the action of an external magnetic field, 6O-MWCNTs@Fe_3_O_4_ that adsorbed metal ions can quickly achieve good separation from the solution. The joint characterization results of FTIR and XPS show that under the action of both coordination and electrostatic attraction, the C=O bond in the –COOH group is induced to open by the metal ions and transforms into an ionic bond, and the metal ions are stably adsorbed on the surface of 6O-MWCNTs@Fe_3_O_4_. Pb(II) has a stronger attraction than Cu(II) and Cd(II) to the lone pair of electrons in oxygen atoms to form complexes, due to the covalent index of Pb (6.41) is more larger than that of Cu (2.98) and Cd (2.71).These data provide a new type of recyclable adsorbent for the efficient treatment of heavy metal ions in wastewater and enrich relevant theoretical knowledge.

## Introduction

With the rapid development of urbanization, industrialization, and agricultural activities, as an important medium for energy conversion and material migration, water resources are inevitably polluted by thousands of toxic and harmful substances produced in various ways. Pollutants in the water environment are roughly divided into two categories: inorganic pollutants and organic pollutants. Heavy metal pollution is a typical inorganic pollutant. Most heavy metals exist in a dissolved ion state and easily accumulate in the environment and organisms, which may cause serious hidden, lagging, and persistent compound pollution in the atmosphere, water, and soil environment^1–3^. Therefore, heavy metal pollution has become a major threat to human health and social development. How to more efficiently treat heavy metal ions in wastewater has become an important environmental issue.

Many methods have been reported to remove heavy metal ions from wastewater, such as chemical precipitation^4^, coagulation^5^, ion exchange^6^, membrane filtration^7–9^, and electrochemical treatment^10,11^, etc. These methods have been used in many industries with good effects, but all of them have their own shortcomings. Chemical precipitation may increase the difficulty of recycling metal sludge generated after wastewater treatment. Ion exchange occurs when the adsorption of heavy metal ions by resin is easily restricted by other variable factors. These limitations have severely affected the large-scale application of ion exchange resins in wastewater treatment. Membrane filtration can reduce the amount of chemicals used and the output of metal sludge, but the maintenance cost is too high, the membrane replacement cycle is short, and the waste membrane pollutants generated need further disposal. Electrochemical wastewater technology requires a relatively large capital investment and expensive power supply, and the low purification efficiency is gradually unable to meet the needs of the industry, making it difficult to achieve wide-ranging applications. Carbon nanotubes (CNTs) have attracted great attention in the wastewater purification of heavy metal ions due to their large specific surface area, small size, hollow, layered structure, etc. Li and Wang^12,13^ found that the adsorption capacity of prepared aligned carbon nanotubes (ACNTs) for fluoride ions could reach more than 4.5 mg/g, when studing the adsorption and removal of inorganic F^−^ anions by CNTs. Liu^14^ used carbon nanotube/calcium alginate (CNTs/CA) composites to remove copper in aqueous solution. When the equilibrium concentration of copper ions reached 5 mg/L, the best adsorption capacity of CNTs/CA for copper ions was 67.9 mg/g. Li^15^ studied the effect of the morphology of oxidized carbon nanotubes (OCNTs) on the removal of Pb(II). The results showed that the adsorption capacity of OCNTs for Pb(II) was closely related to their morphology, and the adsorption capacity of CNTs after oxidation was significantly better than that of CNTs because there were fewer defects. Peng^16^ used carbon nanotube-iron oxide magnetic composites as adsorbents for the removal of Pb(II) and Cu(II) from water, and the maximum adsorption capacities were 0.51 and 0.71 mmol/g, respectively. Pillay^17^, Gupta^18^ and Huang^19^ studied the adsorption capacity of three adsorbents, activated carbon, modified multi-walled carbon nanotubes (MWCNTs) and unfunctionalized MWCNTs, to remove low concentrations of Cr(VI) and found that the adsorption capacity of the unfunctionalized MWCNTs was the best, while the adsorption effect of activated carbon was the worst. Alok Mittal^20^ prepared MWCNTs/ThO_2_ nanocomposites and removed Pb(II) metal from aqueous media. Visibly, CNTs have high efficiency in adsorbing and removing metal ions, but they are difficult to separate from aqueous solutions due to their small size, and are easily discharged into the water environment, causing secondary pollution^21^. Therefore, how to quickly and conveniently solve the problem of difficult separation of CNTs from adsorbed heavy metals urgently needs to be further researched.

In this paper, MWCNTs were first partially oxidized by wet chemicals to load oxygen atoms on the surface to form 6O-MWCNTs, and then recombined with magnetic ions to give them certain supermagnetic properties. Through modification, a new type of adsorbent, namely, 6O-MWCNTs@Fe_3_O_4_, could be obtained and used to remove heavy metals in wastewater. This adsorbent enhanced the adsorption capacities of CNTs for heavy metal ions. Meanwhile, the modified CNTs had certain magnetic properties, which were beneficial to the subsequent magnetic separation of CNTs and the recovery of metal resources. First, the surface of CNTs was modified by the wet chemical oxidation method, and later, further processed by solvothermal methods to prepare 6O-MWCNTs@Fe_3_O_4_ with excellent heavy metal adsorption performance. The adsorption performance was checked through multiple factor comparative experiments. Then, the adsorption mechanism and microstructural changes and were studied in-depth through the joint analysis methods of Fourier-transform infrared spectroscopy (FTIR), X-ray photoelectron spectroscopy (XPS), and X-ray diffraction (XRD), and physical property measurement system (PPMS). Changes in the microscopic morphology of 6O-MWCNTs before and after modification were analyzed by scanning electron microscope and transmission electron microscope (SEM-TEM). These data provide a new type of recyclable adsorbent for the efficient treatment of heavy metal ions in wastewater and enrich relevant theoretical knowledge.

## Experimental and analytical methods

### Materials

The MWCNTs (> 97 wt. %) used in the experiment were supplied by the Chinese Academy of Sciences Chengdu Organic Chemistry Co., Ltd.. Other analytical reagents, such as sulfuric acid (H_2_SO_4_, 98 vol. %), nitric acid (HNO_3_, 65 vol. %), ethylene glycol (C_2_H_6_O_2_), absolute ethanol (C_2_H_6_O), ferric chloride hexahydrate (FeCl_3_·6H_2_O, 99 wt. %), sodium acetate (CH_3_COONa·3H_2_O, 99 wt. %), and sodium hydroxide (NaOH, 99 wt. %) were provided by Sinopharm Chemical Reagent Beijing Co., Ltd.. The mixed solution containing Cu(II), Zn(II), Cd(II), Ni(II) and Pb(II) metal ions used in the adsorption experiment was configured according to the corresponding standard and the concentration requirements. The initial ion concentration of the Pb(II) solution used in the adsorption experiment was 50 mg/L.

### Modification of functional groups on the surface of 6O-MWCNTs

1. Preparation of 6O-MWCNTs
  One gram of MWCNTs was accurately weighed, and thoroughly mixed with 12 ml of concentrated HNO_3_ (65 vol. %) and 36 ml of H_2_SO_4_ (98 vol. %) in a 100 ml three-necked round-bottom flask. The mixture was heated to 75 °C and stirred for 6.0 h to obtain 6O-MWCNTs. To remove the unreacted impurities on the surface of the modified MWCNTs, the obtained materials were purified in two steps with deionized water and absolute ethanol: (1) The mixed products were washed with deionized water and absolute ethanol three times each, ultrasonicated for 30 min, centrifuged at 10,000 r/min for 10 min. (2) The supernatant was removed, and the bottom black powder was vacuum-dried for 12.0 h to obtain the surface modified materials, namely, 6O-MWCNTs. Finally, the product was ground, sieved, sealed, and used as the raw material to load magnetic nanoparticles to prepare 6O-MWCNTs@Fe_3_O_4_.
2. Grafting magnetic nanoparticles on the surface of 6O-MWCNTs
  FeCl_3_·6H_2_O (1.73 g) was weighed and mixed with C_2_H_6_O_2_ (35 ml) in a 100 ml glass beaker, which was stirred until the solid particles completely dissolved^22–24^. Subsequently, 3.83 g of CH_3_COONa·3H_2_O was added, stirred and reacted for 30 min to obtain nanomagnetic particles (Eqs. 1–2). Then, 1 g of 6O-MWCNTs was added to the mixture, and stirred for 30 min. Later, the mixed solution was transferred to a 100 ml polytetrafluoroethylene lined stainless steel autoclave, reacted at 200 °C for 8.0 h, and then naturally cooled to room temperature (Eq. 3). The product was washed repeatedly with deionized water and absolute ethanol. Finally, the magnetic black solid was separated by an external magnet, and the separated black powder was placed in a vacuum drying oven at 80 °C for 12.0 h to obtain 6O-MWCNTs@Fe_3_O_4_.
  1 $${\text{FeCl}}_{{3}} \cdot{\text{6H}}_{{2}} {\text{O}}_{{({\text{s}})}} + {\text{ 3CH}}_{{3}} {\text{COONa}}_{{({\text{s}})}} + {\text{ C}}_{{2}} {\text{H}}_{{6}} {\text{O}}_{{{2}({\text{l}})}} \to {\text{ Fe}}\left( {{\text{OH}}} \right)_{{{3}({\text{s}})}} + {\text{ 3NaCl}}_{{({\text{s}})}} + {\text{3CH}}_{{3}} {\text{COOH}}_{{({\text{l}})}} + {\text{ C}}_{{2}} {\text{H}}_{{6}} {\text{O}}_{{{2}({\text{l}})}} + {\text{ 3H}}_{{2}} {\text{O}}_{{({\text{l}})}}$$
  2 $${\text{Fe}}\left( {{\text{OH}}} \right)_{{{3}({\text{s}})}} + {\text{ C}}_{{2}} {\text{H}}_{{6}} {\text{O}}_{{{2}({\text{l}})}} \to {\text{ Fe}}\left( {{\text{OH}}} \right)_{{{2}({\text{s}})}} + {\text{ C}}_{{2}} {\text{H}}_{{4}} {\text{O}}_{{({\text{l}})}} + {\text{ H}}_{{2}} {\text{O}}_{{({\text{l}})}} + {\text{ OH}}^{ - }_{{({\text{aq}})}}$$
  3 $${\text{2Fe}}\left( {{\text{OH}}} \right)_{{{3}({\text{s}})}} + {\text{ Fe}}\left( {{\text{OH}}} \right)_{{{2}({\text{s}})}} + {\text{ 6O}} - {\text{MWCNTs }} \to {\text{6O}} - {\text{MWCNTs}}@{\text{Fe}}_{{3}} {\text{O}}_{{{4}({\text{s}})}} + {\text{ 4H}}_{{2}} {\text{O}}_{{({\text{l}})}}$$

### Adsorption performance experiment

6O-MWCNTs@Fe_3_O_4_ (20 mg) and 100 ml of the metal ion preparation solution were measured and placed into a 250 ml conical flask. The reaction was shaken at 25 °C and 180 rpm for 60 min. After adsorption reached equilibrium, the mixed liquid was filtered and diluted. Then, the concentration of the heavy metal ions was measured using atomic absorption spectrometry (AAS), and the adsorption capacity was calculated by using the following formula:

$${\mathrm{Q}}_{\mathrm{e}}=\frac{{\mathrm{C}}_{0}\mathrm{V}-{\mathrm{C}}_{\mathrm{e}}\mathrm{V}}{\mathrm{M}}$$

${\mathrm{Q}}_{\mathrm{e}}(\mathrm{mg}/\mathrm{g})$: adsorption capacity; ${\mathrm{C}}_{0}$(mg/l): the initial concentrations of the metal ion preparation solution; ${\mathrm{C}}_{\mathrm{e}}$(mg/l): the equilibrium concentrations of of metal ion; $\mathrm{V}(\mathrm{ml})$: volume of the metal ion configuration solution; $\mathrm{M}(\mathrm{g})$: the adsorbent dose of 6O-MWCNTs@Fe_3_O_4_.

### Analytical methods

The phase composition and crystal form of samples were determined by using XRD (D/max2550 VB) + X with Cu radiation (40 kV, 300 mA); data were collected with a step size of 10°/min for 2θ values ranging from 10° to 80°^25,26^. The concentration of each heavy metal ion in the solution before and after the reaction was determined by AAS (ContrAA 700, Jena Instrument Co., Ltd., Germany). The surface chemistry of samples was studied by XPS with a Thermo Scientific ESCALAB 250Xi spectrometer using an Al Kα X-ray source (1486.6 eV). To compensate for the charging effects, all of the spectra were calibrated with graphitic carbon as a reference at a binding energy (BE) of 284.8 eV. The O1s spectra were deconvolved with the subtraction of linear background and with a Gaussian (80%)-Lorentzian (20%) mixed function. Magnetic properties were detailed by physical property measurement system (PPMS, Quantum Design, USA) at room temperature. FTIR spectra were collected in the range of 400–2000 cm^−1^ using a Nicolet IS10 spectrometer with 4 cm^−1^ resolution. The micromorphology and microstructures changes were observed by SEM and TEM (SEM, JEOL JSM-6360LV instrument; TEM, Philips TECNAI-20, Netherlands).

## Results and discussion

### Characterization of 6O-MWCNTs@Fe_3_O_4_

#### XRD analysis

**Figure 1:**
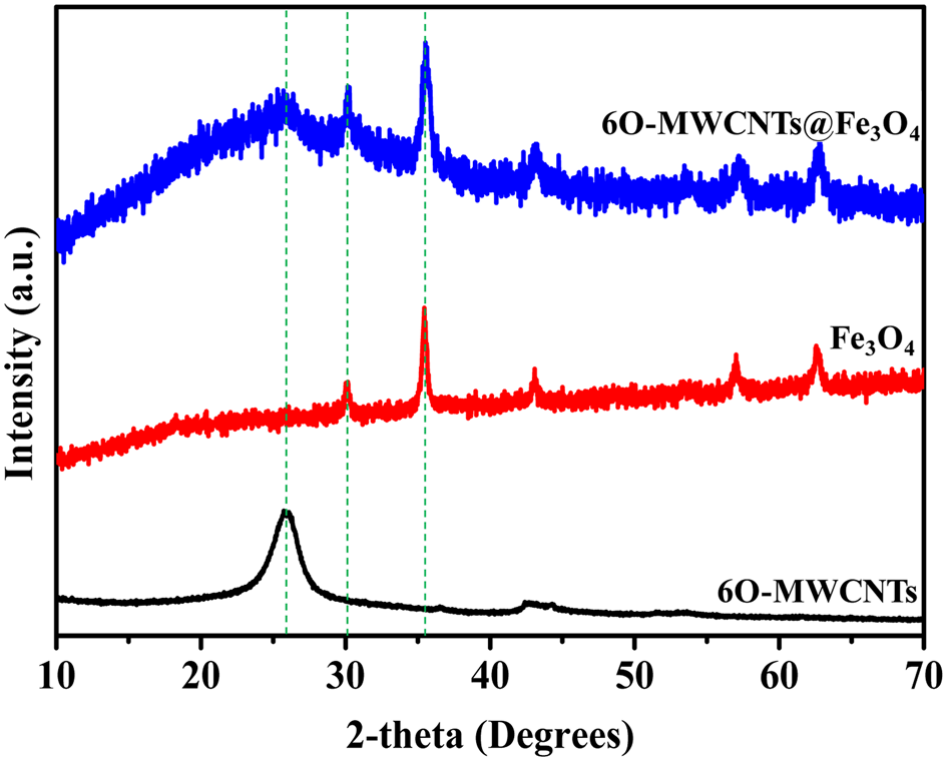
XRD patterns of 6O-MWCNTs, Fe_3_O_4_ and 6O-MWCNTs@Fe_3_O_4_.

XRD was used to resolve the phase and crystal changes of 6O-MWCNTs after magnetic grafting, and the results are shown in Fig. 1. 6O-MWCNTs have two typical characteristic peaks, which are located at 25.9° and 43.6°, and are consistent with those reported in the literature^19^. However, after grafting magnetic nanoparticles onto the surface of 6O-MWCNTs, the peak of 6O-MWCNTs weakens in intensity, and the characteristic peak corresponding to Fe_3_O_4_ appears. The background noise peak of 6O-MWCNTs@Fe_3_O_4_ is obviously more intense than that of 6O-MWCNTs, indicating that the Fe_3_O_4_ on grafted the surface of 6O-MWCNTs is basically in an amorphous state. The figure shows that Fe_3_O_4_ and 6O-MWCNTs coexist in the magnetic MWCNTs.

#### BET analysis

The textural characteristics of the 6O-MWCNTs are shown in Figure S1. In the low-pressure section, the adsorption capacity of the 6O-MWCNTs adsorbent increases slightly, but there is a sudden increase in the adsorption capacity at approximately *p/p*_*0*_ = 0.6 ~ 0.8. The position of this segment can reflect the pore size of the sample and can be used as a basis for measuring the uniformity of the mesopores. In addition, at higher *p/p*_*0*_ vaules, the desorption isotherm does not coincide with the adsorption isotherm, and the desorption isotherm is above the adsorption isotherm. According to the type of mesoporous hysteresis loop^27^, the curve is identified as an H3 type hysteresis loop, and the existence of a hysteresis loop indicates that MWCNTs form a large number of mesopores after partial oxidation, which is conducive to the formation of more adsorption sites.

#### Magnetic property

**Figure 2:**
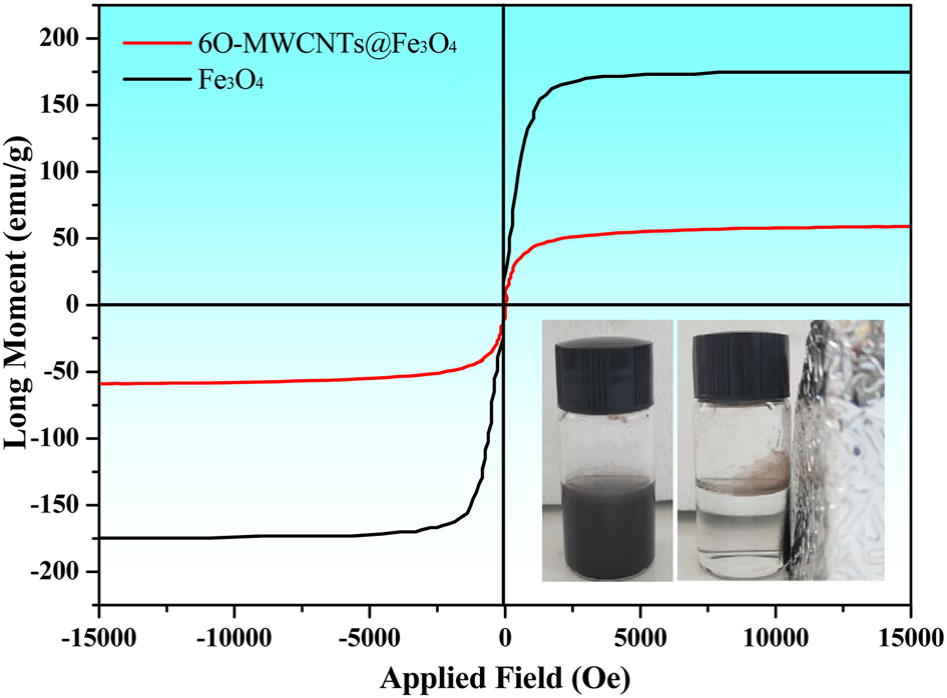
Changes in the hysteresis loop of 6O-MWCNTs loaded with magnetic ions.

The change in the magnetic property of 6O-MWCNTs after grafting with magnetic particles is shown in Fig. 2. As indicated in Fig. 2, there is almost immeasurable remanence and coercivity, which demonstrate that pure Fe_3_O_4_ and 6O-MWCNTs@Fe_3_O_4_ show typical superparamagnetic behavior. Moreover, the saturation magnetizations of the as-prepared Fe_3_O_4_ and 6O-MWCNTs@Fe_3_O_4_ were 176.01 emu/g and 59.46 emu/g, respectively, as estimated using PPMS at room temperature. This saturation magnetization of 6O-MWCNTs@Fe_3_O_4_ is lower than that of pure Fe_3_O_4_, which may be attributed to the impact of macromolecules and carbon-based materials in composites. Generally, nanoparticles with saturation magnetizations of 16.3 emu/g could be separated magnetically from solution using a magnet. Thus, the 6O-MWCNTs@Fe_3_O_4_ prepared in this study are easily separated from aqueous solutions, and can possibly be ideal adsorbents and carrier. 6O-MWCNTs@Fe_3_O_4_ can achieve high-efficiency separation through an external magnetic field after selective adsorption of lead ions.

#### FTIR analysis

**Figure 3:**
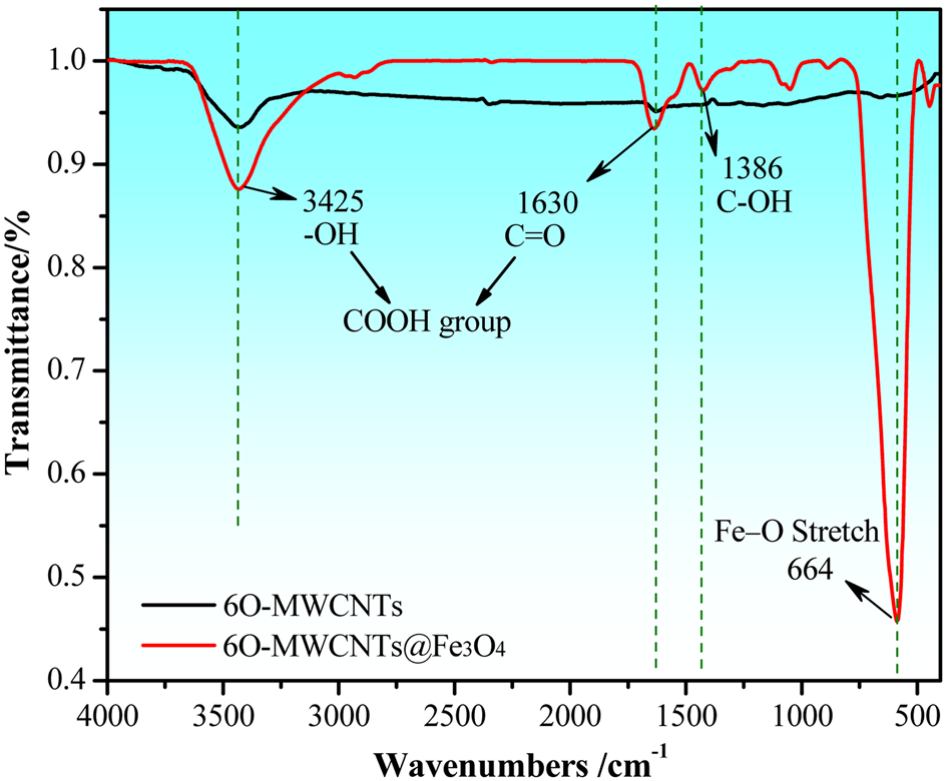
FTIR spectras of 6O-MWCNTs and 6O-MWCNTs@Fe_3_O_4_.

To further investigate the changes in the surface functional groups of 6O-MWCNTs, FTIR was used to compare and analyze the samples after grafting, as shown in Fig. 3. After grafting magnetic particles on the surface of 6O-MWCNTs, the positions of the peak corresponding to the functional groups on 6O-MWCNTs are basically unchanged, but the peak intensity is significantly enhanced. The strong peak at 3425 cm^−1^ is assigned to the -OH stretching mode of the COOH group^28,29^, while the characteristic absorption peak located at 1630 cm^−1^ is assigned to the C=O stretching of the COOH group^30,31^. The characteristic absorption peak at 1386 cm^−1^ is attributed to C–OH stretching^32,33^. In particular, a new peak appearing at 664 cm^−1^ is caused by Fe–O bond tensile vibrations^34,35^. According to the comparison results, the formed 6O-MWCNTs@Fe_3_O_4_ not only retains the inherent functional groups (C=O, –OH, and C–OH) of 6O-MWCNTs, but also presents new bonds (Fe–O) corresponding to the magnetic particles.

#### SEM-TEM analysis

**Figure 4:**
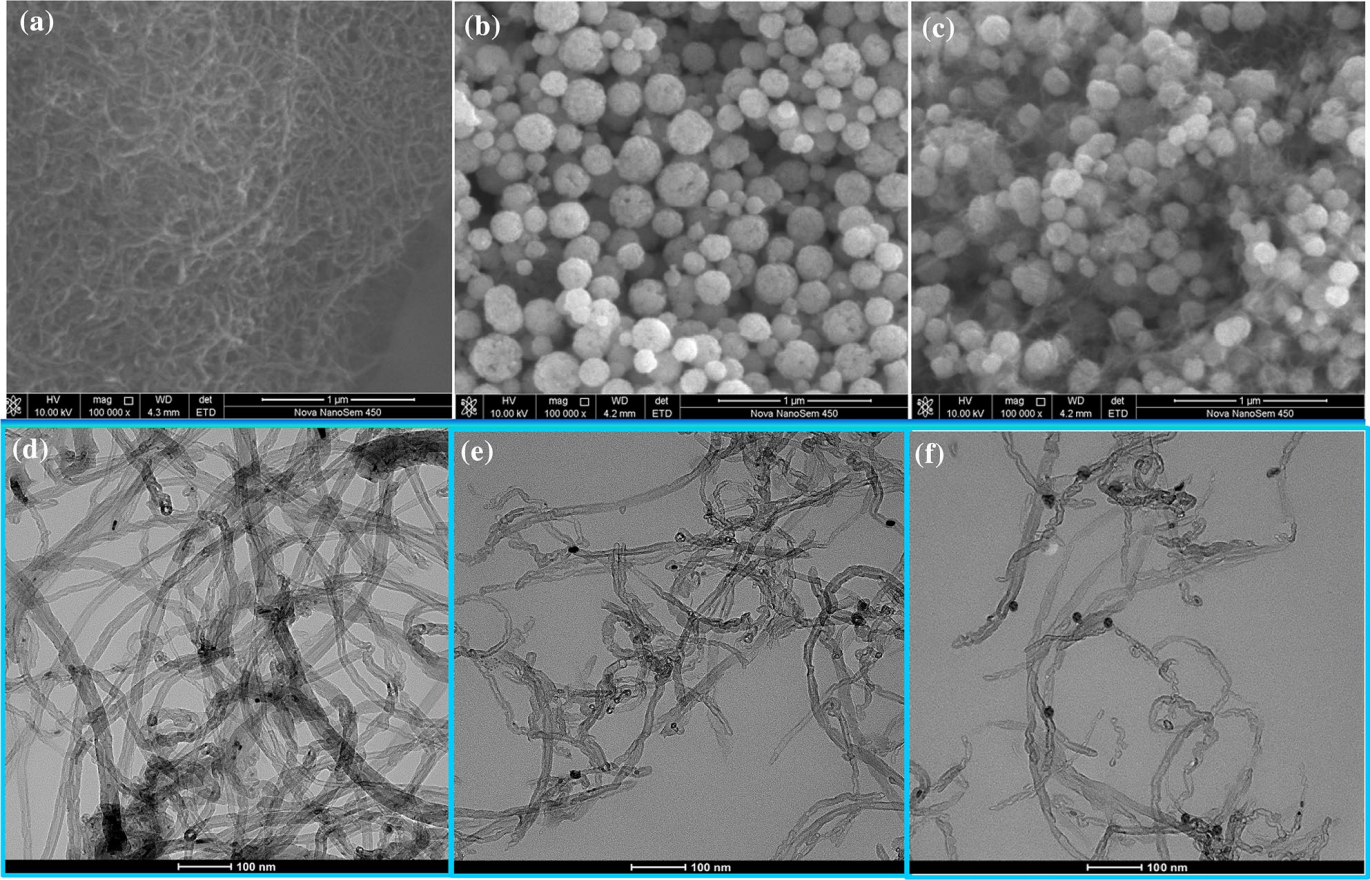
SEM images of 6O-MWCNTs (**a**), pure Fe_3_O_4_ (**b**), and 6O-MWCNTs@Fe_3_O_4_ (**c**) and TEM images of MWCNTs (**d**), 6O-MWCNTs (**e**), and 6O-MWCNTs@Fe_3_O_4_ (**f**).

The generation of 6O-MWCNTs@Fe_3_O_4_ is further proven by the differences and changes in the microscopic morphologies of the product and raw material (shown in Fig. 4). 6O-MWCNTs have a typical tubular structure with a small diameter, and the tubes will entangle with each other to form a similar fishnetlike structure (Fig. 4a). Fe_3_O_4_ nanoparticles show a quasispherical morphology, and their sizes are almost uniformly distributed (Fig. 4b). After mixing with magnetic particles, a large number of spherical particles appear on the surface of the 6O-MWCNTs (Fig. 4c). Tubular 6O-MWCNTs are similar to a needle thread connecting magnetic particles in series. In addition, TEM images of 6O-MWCNTs and 6O-MWCNTs@Fe_3_O_4_ are shown in Fig. 4. Before the 6O-MWCNTs are loaded with magnetic ions, the shape of the MWCNTs and 6O-MWCNTs had an irregular line-like morphology with a smooth surface (Fig. 4d, e). However, interestingly, after the intervention of magnetic particles, many quasi-spherical granules with an average particle size of 20 nm are unevenly distributed on the surface of the carbon nanotubes (Fig. 4f). These results further demostrate that Fe_3_O_4_ and 6O-MWCNTs coexist in 6O-MWCNTs@Fe_3_O_4_.

### Adsorption performance

1. Effect of pH on the adsorption performance
  The adsorption performance of 6O-MWCNTs@Fe_3_O_4_ on metal ions at different pH is shown in Fig. 5. Before grafting magnetic particles on the surface of 6O-MWCNTs, a certain adsorption effect on Pb(II), Cu(II) and Cd(II) is observed, among which the adsorption effect on Pb(II) is the best. The maximum adsorption capacity is 96.09 mg/g, when pH = 6 (Fig. 5a). However, after grafting, with the other conditions unchanged, as the pH of the solution increases, the adsorption capacities of 6O-MWCNTs@Fe_3_O_4_ for the three metal ions all present a trend of first increasing (pH ≤ 6), and then decreasing (pH > 6). The adsorption effect on Pb(II) is much better than that on the other two metal ions, with the maximum adsorption capacity reaching 205.8 mg/g (Fig. 5b), which is much higher than the existing adsorption capacity of the same type of adsorbents, while the adsorption capacities for Cu(II), and Cd(II) are only 87.1 mg/g, 57.3 mg/g, respectively. The reason for this may be related to the dissociation of the carboxyl group. H^+^ inhibits the dissociation of carboxyl groups and hinders the formation of O– and COO–^36^. The increase in pH means that the H^+^ content in the solution decreases, which will cause the protonation effect of the carboxyl groups on the surface of 6O-MWCNTs@Fe_3_O_4_ to weaken, and the number of adsorption sites on the surface of 6O-MWCNTs@Fe_3_O_4_ increases, making more carboxyl groups coordinate with metal ions; therefore, the saturation adsorption capacity increases. However, as the pH further increases, the solution gradually becomes alkaline. According to the metal ion dissolution precipitation curve, when pH > 5, although the metal ions in the solution are still mainly adsorbed, some metal ions have begin to precipitate. As the pH continues to increase, the precipitation trend further intensifies, which is also the reason for the sharp decline in the adsorption effect of 6O-MWCNTs@Fe_3_O_4_.

**Figure 5:**
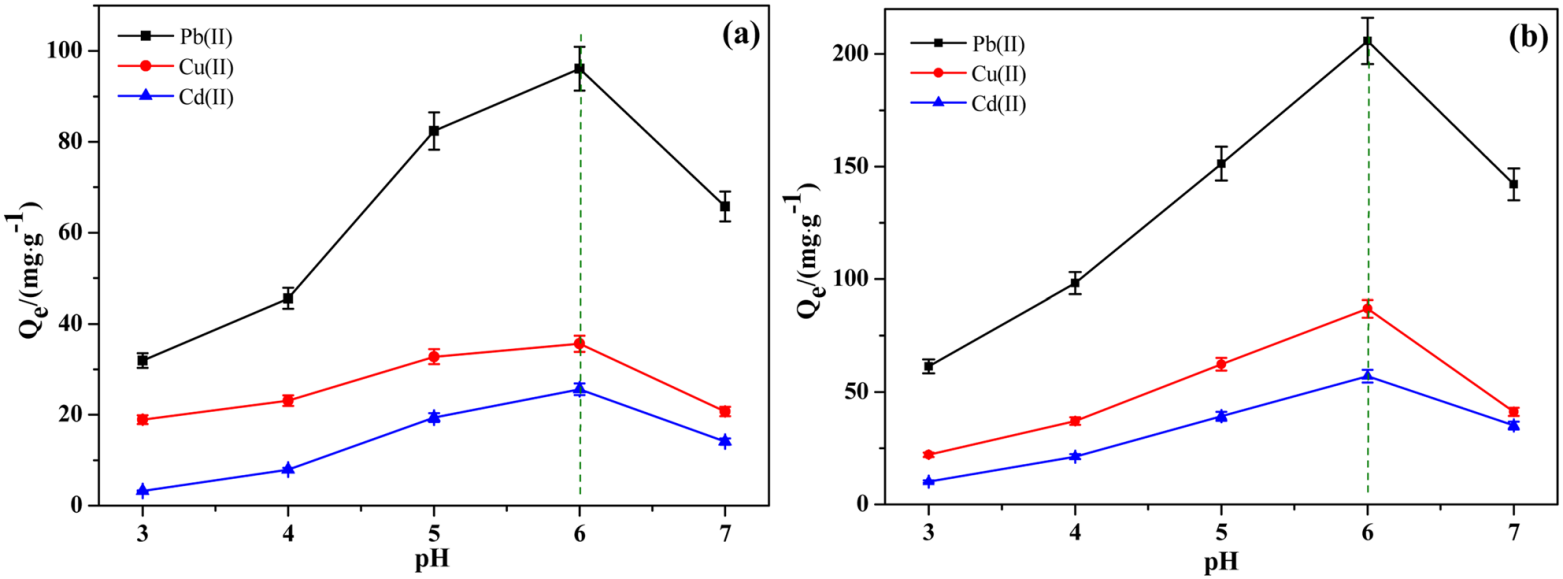
Effect of pH on the adsorption performance of 6O-MWCNTs (**a**) and 6O-MWCNTs@Fe_3_O_4_ (**b**).
2. Effect of the initial concentration of metal ions
  The effect of the initial concentration (*C*_*0*_) of different metal ions on the adsorption performance of 6O-MWCNTs@Fe_3_O_4_ is shown in Fig. 6. Although the metal ion adsorption curves of 6O-MWCNTs@Fe_3_O_4_ and 6O-MWCNTs have similar trends, the adsorption capacity of 6O-MWCNTs@Fe_3_O_4_ is much higher than that of 6O-MWCNTs (Fig. 6a). As the initial concentration of metal ions in the solution increases, the adsorption capacities of 6O-MWCNTs@Fe_3_O_4_ for various metal ions shows an overall upward trend (Fig. 6b). After reaching a certain concentration, the upward trend of the adsorption curve slows down. An increase in the concentration of metal ions means that the absolute content of metal ions per unit volume increases. The chance of metal ions contacting 6O-MWCNTs@Fe_3_O_4_ increases, resulting in a rapid increase in the amount of metal ions adsorbed. However, with a further increase in the initial concentration, the adsorption efficiency of 6O-MWCNTs@Fe_3_O_4_ for metal ions reaches saturation, and the growth rate of the adsorption curve becomes flat.

**Figure 6:**
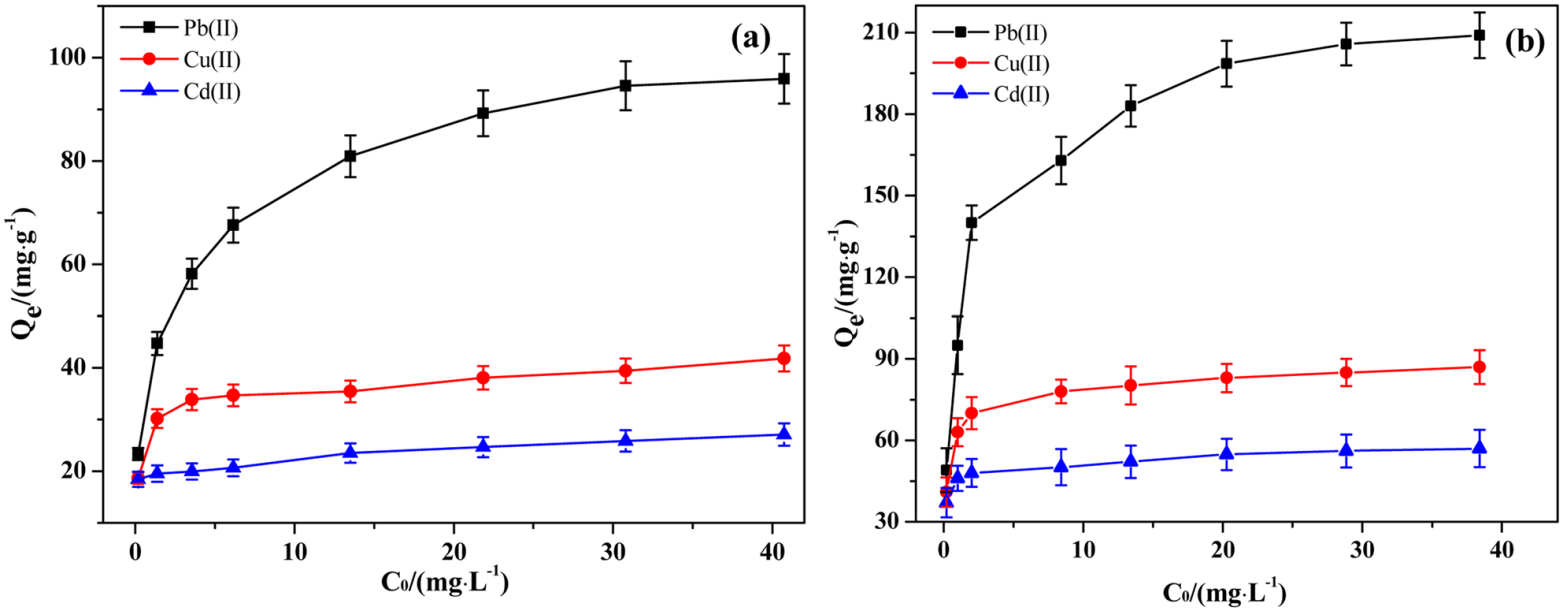
Effect of the initial concentration of metal ions on the adsorption performance of 6O-MWCNTs (**a**) and 6O-MWCNTs@Fe_3_O_4_ (**b**).
3. Effect of adsorption time
  The effect of the adsorption time on the adsorption performance of 6O-MWCNTs@Fe_3_O_4_ with the other factors being constant is shown in Fig. 7**.** The adsorption capacity of Pb(II), Cu(II), and Cd(II) increases rapidly, within 10 min. With a further increase of the adsorption time, although the adsorption capacity continues to increase, the increase is obviously weakened. After 30 min, the adsorption reaction basically reaches equilibrium. The reason for this is related to the adsorption sites on the surface of the adsorbent. In the initial stage, there were enough adsorption sites on the surface of 6O-MWCNTs@Fe_3_O_4_, and the concentration of metal ions was high. Coordination between the adsorption sites and the metal ion has a high success rate, leading to a rapid increase in the adsorption rate in the early stage. However, as the number of adsorption sites decreases, the adsorption capacity of carbon nanotubes is close to saturation. When the adsorption time is prolonged, the absolute adsorption capacity is basically not affected, and the adsorption curve is basically a horizontal line.

**Figure 7:**
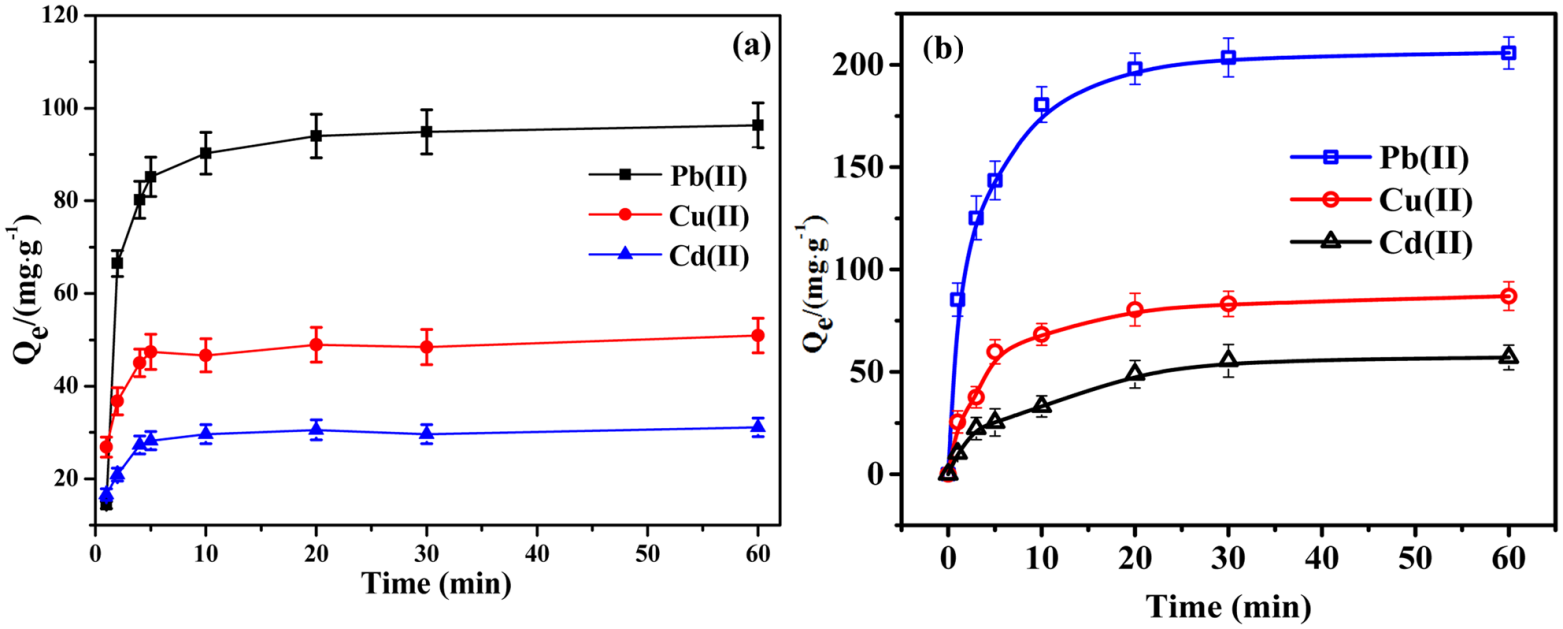
Effect of adsorption time on the adsorption performance of 6O-MWCNTs (**a**) and 6O-MWCNTs@Fe_3_O_4_ (**b**).
4. Effect of coexisting metal ions
  To investigate the adsorption performance of 6O-MWCNTs@Fe_3_O_4_ in the solution of coexisting metal ions, the carbon nanotubes were mixed with a solution containing Cd(II), Ni(II), Zn(II), Cu(II), and Pb(II) and multiple ions of the same valence for the adsorption reaction, and the result is shown in Fig. 8. The adsorption capacity of 6O-MWCNTs@Fe_3_O_4_ for Pb(II) reaches 92.2 mg/g, while the adsorption capacity of Cd(II), Ni(II), Zn(II), and Cu(II) only reaches 19.3 mg/g, 8.7 mg/g, 6.6 mg/g, and 30.2 mg/g, respectively. The adsorption capacity for Pb(II) is much greater than that forother competing ions. Visibly, under the interference of many same-valent ions, 6O-MWCNTs@Fe_3_O_4_ have a good selective adsorption effect on lead ions.

**Figure 8:**
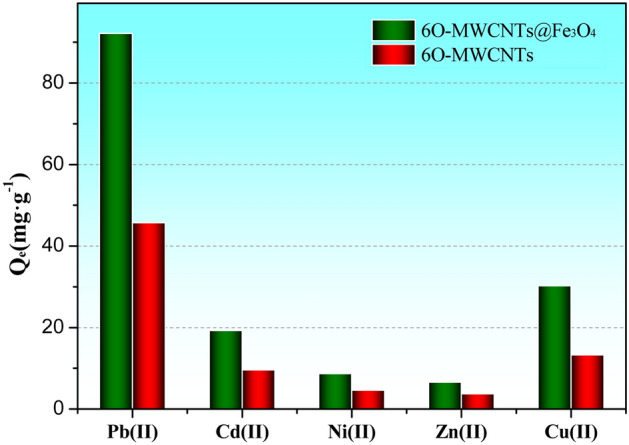
Effect of coexisting metal ions on the adsorption performance of 6O-MWCNTs@Fe_3_O_4_.

### Magnetic separation performance

**Figure 9:**
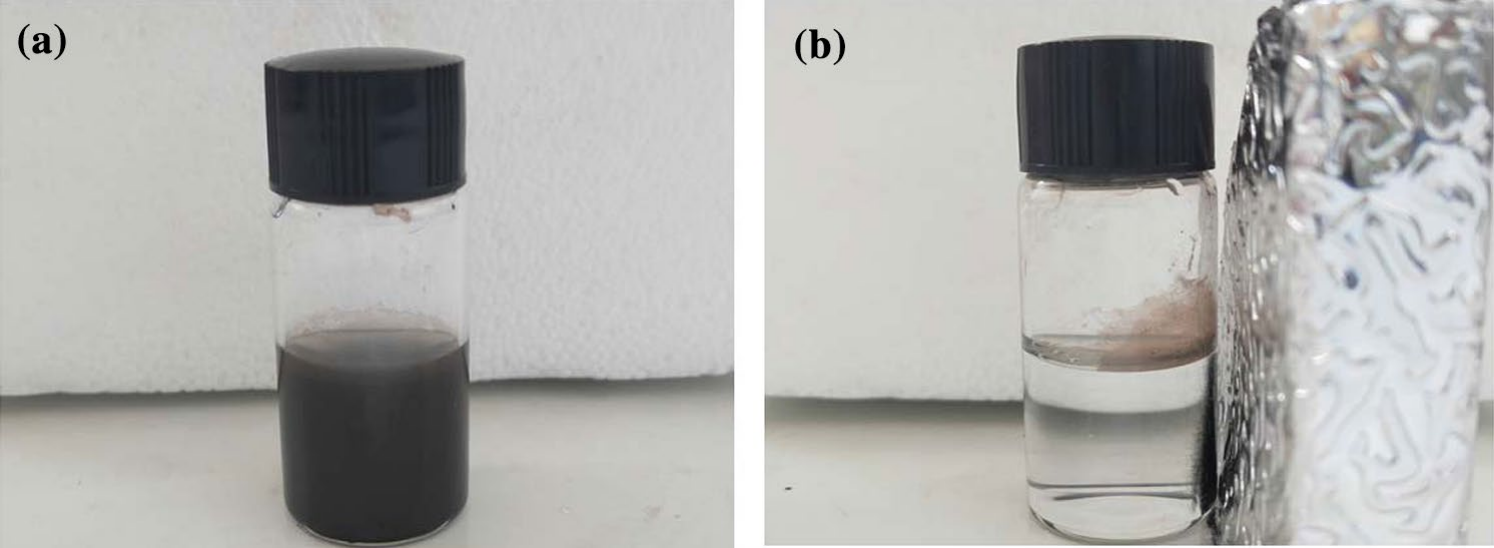
Photo of the 6O-MWCNTs@Fe_3_O_4_ separation and redispersion process. (**a**) without an external magnetic field and (**b**) with an external magnetic field.

To study the magnetic separation effect of 6O-MWCNTs@Fe_3_O_4_ after adsorbing metal ions, a magnetic field was gradually applied to 6O-MWCNTs@Fe_3_O_4_ saturated with adsorbed metal ions. The comparison results are shown in Fig. 9. After 6O-MWCNTs@Fe_3_O_4_ adsorbs heavy metals, they are evenly dispersed in the solution to form a suspended turbid liquid when the magnetic field is notapplied. However, the magnetic field gradually approaches, the 6O-MWCNTs@Fe_3_O_4_ with adsorbed heavy metals migrate in the direction of the magnetic field and finally adhere to the wall, and the solution becomes clear. After the external magnetic field moves away, the separated 6O-MWCNTs@Fe_3_O_4_ with adsorbed heavy metals and the clarified liquid will become turbid again. These results show that by applying an external magnetic field, 6O-MWCNTs@Fe_3_O_4_ after the adsorption of heavy metals can be separated quickly from the solutionto recycle metal resources.

### Adsorption mechanism

**Figure 10:**
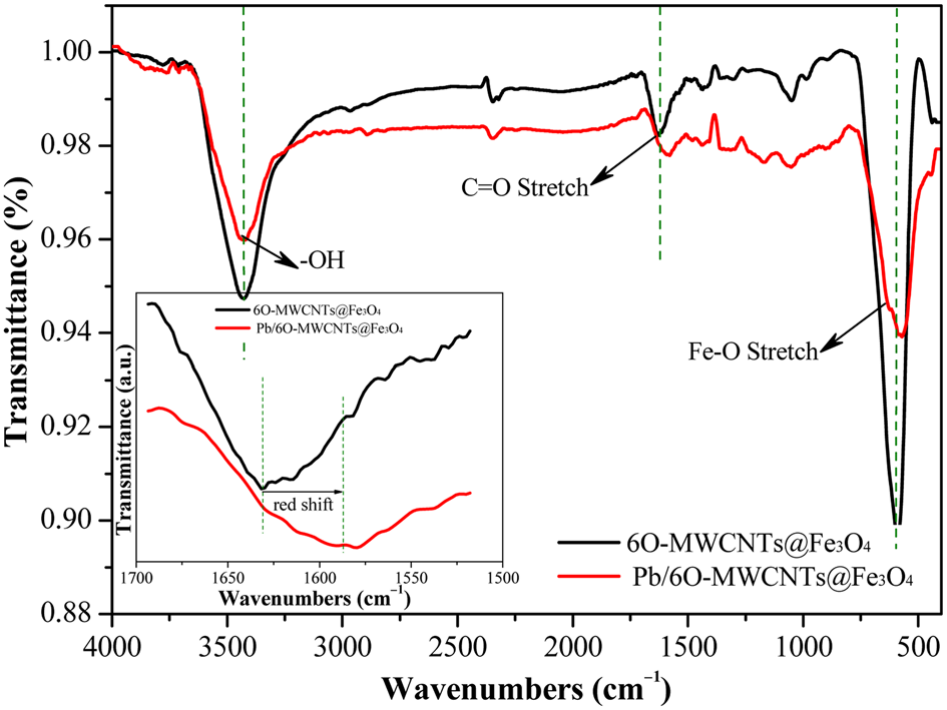
FTIR spectra of 6O-MWCNTs@Fe_3_O_4_ after Pb(II) adsorption.

Based on the previous results, 6O-MWCNTs@Fe_3_O_4_ had good selective adsorption performance in heavy metal-containing wastewater. To ascertain the adsorption mechanism, the changes in functional groups before and after adsorption of Pb(II) were analyzed by FTIR. The comparison result is shown in Fig. 10. After adsorping Pb(II), the intensity of vibration absorption peaks corresponding to the functional groups of –OH and C=O in the COOH group significantly weakens, and the same applies to the Fe–O bond in Fe_3_O_4_. The reason for this is that after adsorbing Pb(II), a new bond forms between the COOH group and Pb(II), resulting in a decrease in the relative amount of COOH. In addition, the characteristic peaks of C=O and Fe–O bonds present a redshift, indicating that the newly formed bond between the COOH group and Pb(II) is more stable than COOH, namely, adsorbed ions can stably exist in the solution. In summary, –OH and C=O bonds on the surface of 6O-MWCNTs@Fe_3_O_4_ may be effective sites for the adsorption of heavy metal ions, and XPS needs to be used to further compare and analyze the changes in the surface chemical state.

**Figure 11:**
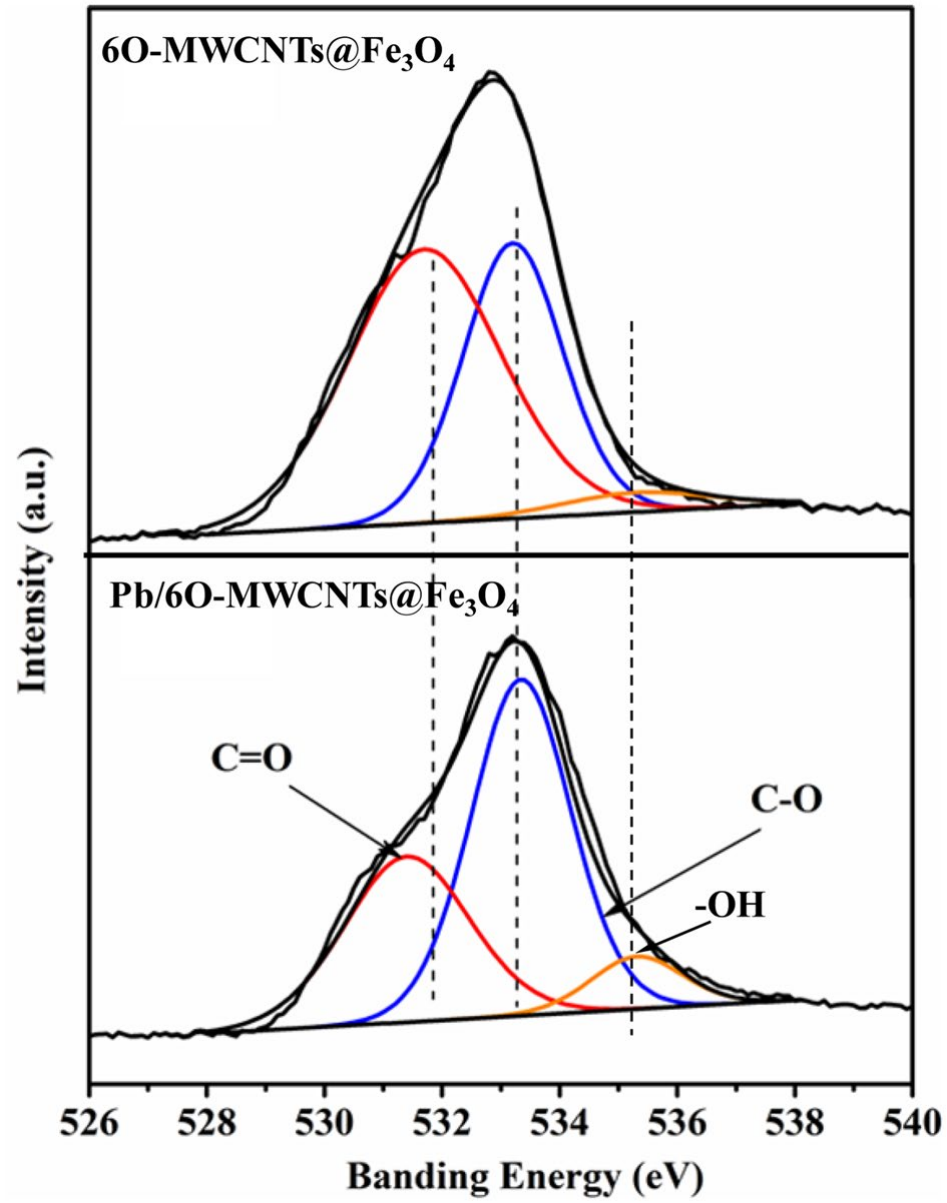
Typical deconvoluted peaks of O1s before and after adsorption of Pb(II).

Almost all the chemical bonds on the surface of 6O-MWCNTs@Fe_3_O_4_ involve oxygen atoms. Therefore, the changes in O1s after Pb(II) adsorption were further analyzed, and the deconvoluted peak results are shown in Fig. 11. The O1s peak is divided into three peaks at 531.47 eV, 533.34 eV, and 535.31 eV, which are assigned to C=O, C–O, and C–OH bonds, respectively^37,38^. According to the literature^37–40^, the O1s binding energies of –OH, C–O, C=O, and O-Pb(II) follow the order –OH > C–O > C=O > O–Pb(II). Therefore, the O1s shift to a lower binding energy area is attributed to the formation of new bonds between the functional groups on the surface of 6O-MWCNTs and Pb(II) through O atoms. In addition, the corresponding relative contents of C=O and –OH bonds decrease from 57.14% and 8.19% to 37.93% and 4.03%, respectively (Table 1), while the content of C–O bonds increases from 34.68% to 58.07%. The ratio of C=O/C–O decreases from 1.65 before adsorption to 0.65, indicating that the C=O bond (belonging to the COOH group) on the surface of 6O-MWCNTs@Fe_3_O_4_ is the main adsorption site for Pb(II). That is, after C=O adsorbs Pb(II), the C=O double bond is opened and converted into a C–O bond and Pb–O bond.

**Table 1:** Peak positions and relative abundance from curve fitting of O1s.

Chemical bonds	6O-MWCNTs@Fe_3_O_4_		Pb/6O-MWCNTs@Fe_3_O_4_	
	BE (eV)	Percentage (%)	BE (eV)	Percentage (%)
C=O	531.47	57.14	531.70	37.93
C–O	533.34	34.68	533.21	58.04
–OH	535.31	8.19	535.3	4.03

**Figure 12:**
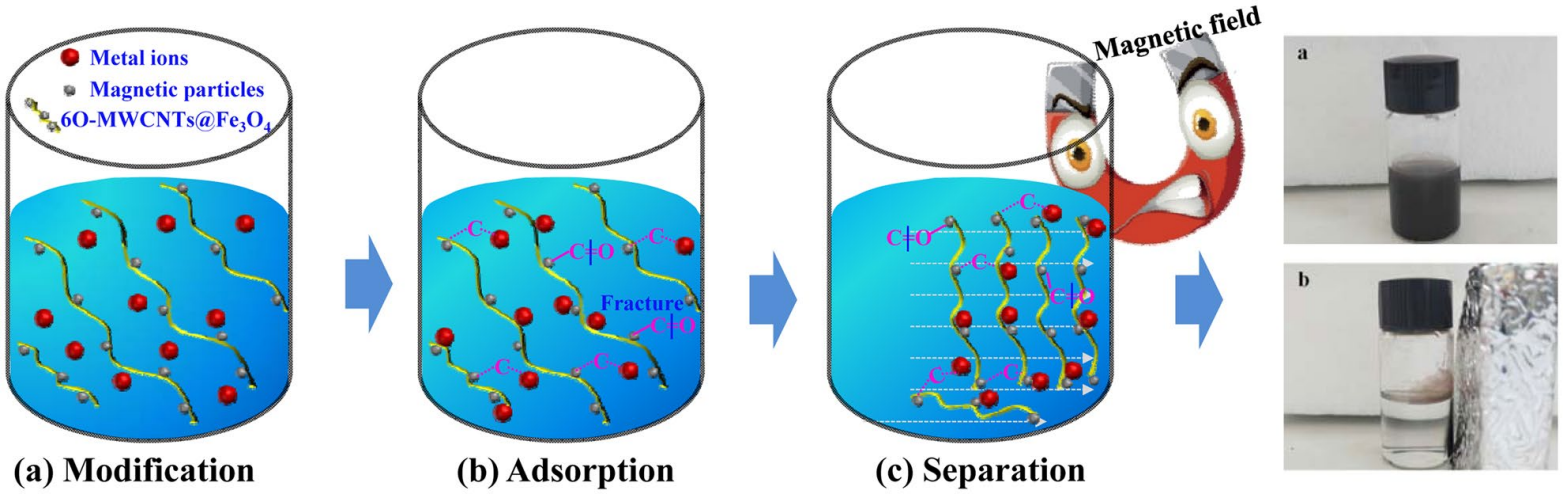
The mechanism of 6O-MWCNTs@Fe_3_O_4_ adsorbing heavy metal ions in wastewater.

Based on the above analysis results, we speculate that the mechanism of 6O-MWCNTs@Fe_3_O_4_ adsorbing heavy metal ions is as follows: when 6O-MWCNTs recombines with magnetic particles, the COOH group will be loaded on the surface of 6O-MWCNTs@Fe_3_O_4_, which is composed of two chemical bonds, i. e., –COOH and –OH bonds (Fig. 12a). According to Chen's research^41^, the O-containing functional groups (-COOH, -OH) are likely located at tube ends and defect sites on the tube sidewalls. As known, Pb(II), Cu(II) and Cd(II) ions have an empty d orbit, oxygen atom has a lone pair of electron that can bind metal ions through electron pair sharing to form the complex^42^. The hydroxyl (–OH) and carboxyl group (-COOH) on 6O-MWCNTs@Fe_3_O_4_ is freely available for characteristic coordination bonding with metal ions^43^. The main binding sites are located at the oxygen atom of carboxyl group on the oxygen-containing functional groups on the 6O-MWCNTs@Fe_3_O_4_ hybrid. C=O bond in the COOH group opens and transforms from covalent bond to an ionic bond, and forms a new C–O–Pb(II) bond (Fig. 12b). The conversion of covalent bonds to ionic bonds is equivalent to O atoms obtaining an electron cloud. This also explains the shift in the O1s peak to a lower binding energy after adsorption of Pb(II). In addition, hydroxyl group and carboxyl group on 6O-MWCNTs@Fe_3_O_4_ surface afford deprotonation reaction under a certain pH value, and the surface charge becomes more negative. The electrostatic attraction may form between the negatively charged functional groups such as COO– bond and O– bond on the surface of 6O-MWCNTs@Fe_3_O_4_ and Pb(II), Cu(II) and Cd(II) ions^44^. Although the pKsp of COO- and Pb(II) is smaller than that of Cu(II), the radius of Pb(II) (0.119 nm)^45^ is much larger than that of Cu(II) (0.073 nm)^46^ and Cd(II) (0.095 nm)^47^. The special pore structure of 6O-MWCNT@Fe_3_O_4_ enables more effective coordination between –COOH, –OH and Pb(II), which results in the adsorption effect of 6O-MWCNT@Fe_3_O_4_ on Pb(II) is far greater than that of Cu(II) and Cd(II) (Fig. 12c).. Therefore, under the action of both coordination and electrostatic attraction, the adsorbent exhibits a high selective adsorption capacity for Pb(II) ion.

### Selective adsorption mechanism

Although both FTIR and XPS studies revealed the adsorption mechanism that Pb(II), Cu(II) and Cd(II) formed complexes with the oxygen atoms of 6O-MWCNTs@Fe_3_O_4_, the mechanism cannot be effectively used to explain the selective adsorption behaviors of Pb(II), Cu(II) and Cd(II) on 6O-MWCNTs@Fe_3_O_4_. Comparing the characteristic properties of Pb(II), Cu(II) and Cd(II), the differences in covalent index (${X}_{m}^{2}r$, where ${X}_{m}$ is electronegativity and $r$ is ionic radius) may be the reason causing the selective adsorption of Pb(II), Cu(II) and Cd(II) by 6O-MWCNTs@Fe_3_O_4_. ${X}_{m}^{2}r$ is a measure for a metal ion of the importance of covalent interactions relative to ionic interactions. According to the Nieboer and Richardson^31^, the larger the ${X}_{m}^{2}r$, the more characteristics of soft acids (HSAB theory), and the metal ions preferentially interacted with the functional group. The covalent index decreased in the following order: Pb (6.41) > Cu (2.98) > Cd (2.71)^48^, suggesting that Pb(II) has a stronger attraction than Cu(II) and Pd(II) to the lone pair of electrons in oxygen atoms to form complexes^49^. Vijayaraghavan et al.^50^ studied the application of sargassum biomass to removal heavy metal ions from synthetic multi-metal (Pb(II), Cu(II), Zn(II) and Mn(II)) solutions, they also confirmed that both electronegativity and ionic radii determine the order of preference of metal binding onto alginate.

The removal capacity of 6O-MWCNT@Fe_3_O_4_ is compared with some of other adsorbents reported in the literature (Table 2). It is seen from the comparison results that the reported different types of adsorbents all have a certain degree of adsorption on metal ions, but the adsorption effect is generally not particularly good. Among them, only γ-PGA-Fe_3_O_4_-GO-(o-MWCNTs) has a maximum adsorption capacity of 574.7 mg/g and 625.0 mg/g for Cu(II) and Cd(II). The 6O-MWCNTs@Fe_3_O_4_ prepared in this manuscript has the largest adsorption capacity for Pb(II), reaching to 215.05 mg/g, indicating that 6O-MWCNTs@Fe_3_O_4_ has the best selective adsorption performance for Pb(II).

**Table 2:** Comparison of the maximum adsorption capacity of Cd(II), Cu(II) and Pb(II) ions on various adsorbents.

Adsorbents	Metal ions	Q_max_(mg/g)	pH	Refs
6O-MWCNTs@Fe_3_O_4_	Pb(II)	215.05	6.0	This work
MWCNTs/ThO_2_	Pb(II)	178.25	5.5	^20^
50%CNTs/Fe_3_O_4_	Pb(II)	40.88	6.2	^51^
CNTs	Pb(II)	102.04	5.0	^52^
CS-MA-DETA	Pb(II)	239.2	5.0	^53^
magnetic chitosan with xanthate (XMCS)	Pb(II)	76.9	5.0	^54^
6O-MWCNTs@Fe_3_O_4_	Cu(II)	87.41	6.0	This work
CNTs/CA	Cu(II)	67.9	2.1	^14^
Fe_3_O_4_ nanoparticles	Cu(II)	35.46	4.0	^55^
magnetic chitosan with xanthate (XMCS)	Cu(II)	34.5	5.0	^54^
γ-PGA-Fe_3_O_4_-GO-(o-MWCNTs)	Cu(II)	574.7	8.0	^56^
6O-MWCNTs@Fe_3_O_4_	Cd(II)	57.18	6.0	This work
Fe_3_O_4_ nanoparticles	Cd(II)	35.46	4.0	^55^
γ-PGA-Fe_3_O_4_-GO-(o-MWCNTs)	Cd(II)	625.0	5.0	^56^
CS-MA-DETA	Cd(II)	201.6	5.0	^53^
Microwave assisted MWCNTs	Cd(II)	88.62	5.0	^57^

## Conclusions

6O-MWCNTs magnetically modified by a solvothermal method, not only inherit their own advantages, such as a large specific surface area and abundant pore structure, but also exhibit a new magnetic properties and further improved adsorption capacity, which is conducive to the magnetic separation and recovery of heavy metals. The XRD, FTIR and SEM results indicate that carboxyl functional groups (–COOH) and magnetic groups (Fe–O) are effectively introduced on the surface of the obtained 6O-MWCNTs@Fe_3_O_4_. Fe_3_O_4_ particles are evenly distributed on the surface of the 6O-MWCNTs structure. Tubular 6O-MWCNTs appear like a needle thread connecting magnetic particles in series, forming a structure similar to a fishing net. The adsorption experiment shows that 6O-MWCNTs@Fe_3_O_4_ have good selective adsorption performance for Pb(II), with a maximum adsorption capacity of 215.05 mg/g, which is much higher than the existing adsorption capacity of the same type of adsorbents. The 6O-MWCNTs@Fe_3_O_4_ after adsorbing metal ions can be separated quickly from solution under the action of an external magnetic field. FTIR and XPS results show that under the action of both coordination and electrostatic attraction, the C=O bond in the –COOH group is induced to open by the metal ions and transforms into an ionic bond, and the metal ions are stably adsorbed on the surface of 6O-MWCNTs@Fe_3_O_4_. Pb(II) has a stronger attraction than Cu(II) and Pd(II) to the lone pair of electrons in oxygen atoms to form complexes, due to the covalent index of Pb (6.41) is more larger than that of Cu (2.98) and Cd (2.71).These data provide a new type of recyclable adsorbent for the efficient treatment of heavy metal ions in wastewater and enrich relevant theoretical knowledge.

## Supplementary Information

Supplementary Information.
