# Supplementary material for: The selective adsorption performance and mechanism of multiwall magnetic carbon nanotubes for heavy metals in wastewater
Source: Sci Rep. 2021 Aug 19;11:16878. doi: 10.1038/s41598-021-96465-7 (PMC8377063; doi:10.1038/s41598-021-96465-7)
Supplement: Supplementary file 1 — Supplementary Information. [file 41598_2021_96465_MOESM1_ESM.pdf]

# The selective adsorption performance and mechanism of multiwall magnetic carbon nanotubes for heavy metals in wastewater

Zhongbing Wang[a, b, d, #], Wenbin Xu[b], Fanghui Jie[c], Zongwen Zhao[a, b, e, #], Kai Zhou[b], Hui Liu[a]

a. School of Metallurgy & Environment, Central South University, 410083, Changsha, Hunan, China

b. Dongjiang Environmental Co., Ltd., 518057, Shenzhen, Guangdong, China

c. Jiangxi Ganchang Evaluation and Testing Technology Consulting Co., Ltd., 330063, Nanchang, Jiangxi, China

d. Postdoctoral mobile Station of Central South University, 410083, Changsha, Hunan, China

e. Shandong Humon Smelting Co., Ltd, 264109, Yantai, Shandong, China

# Corresponding author: Zhongbing Wang, wzbing@126.com;

Zongwen Zhao, zhaozongw@126.com.

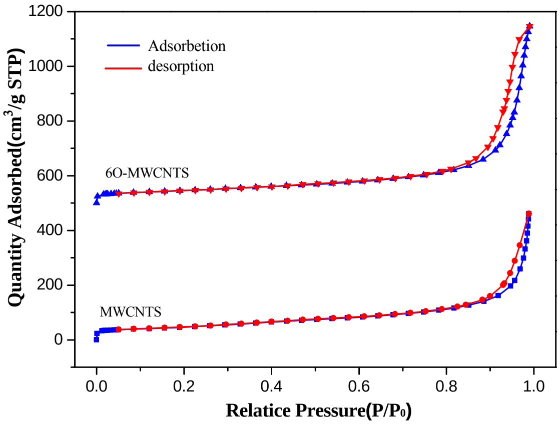


Fig. S1 BET results of 6O-MWCNTs and MWCNTs (Surface area is measured $N_2$ adsorption at 77 K)

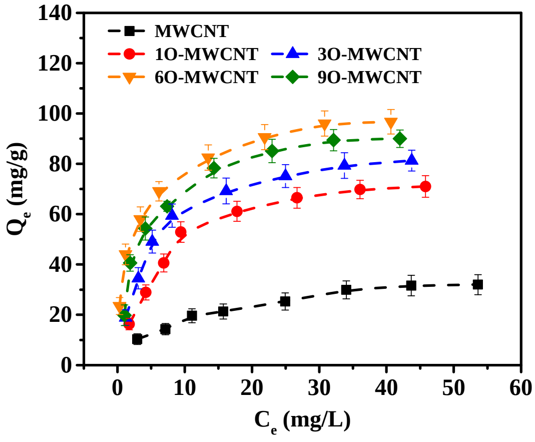


Fig. S2 The adsorption capacity of MWCNTs with different degrees of surface functionalization for Pb(II)

## 1.1 Different adsorption isotherms

To further explore the key control factors in the adsorption process. On the basis of obtaining the influence curve of adsorption time, the quasi-first-order kinetics, quasi-second-order kinetic equations and intra-particle diffusion model are used to fit and analyze the adsorption kinetics data, respectively. The calculation equations of pseudo-first-order dynamics and pseudo-second-order dynamics are as follows:

Pseudo-first-order model:

$$In(Q_e - Q_t) = InQ_e - K_1 t \quad \text{(Eq. 1)}$$

Pseudo-second-order model:

$$\frac{t}{Q_t} = \frac{1}{K_2 {Q_e}^2} + \frac{t}{Q_e} \quad \text{(Eq. 2)}$$

Intra-particle diffusion model:

$$Q_t = K_i t^{\frac{1}{2}} + C \quad \text{(Eq. 3)}$$

$Q_t$ (mg/g) refers to the amount of ion adsorption at time t (min);

$Q_e$ (mg/g) refers to the amount of ion adsorption at equilibrium;

$K_1$ ($min^{-1}$) represents the rate constant of the quasi-first-order equation;

$K_2$ ($g \cdot mg^{-1} \cdot min^{-1}$) represents the rate constant of the quasi- second -order equation;

$K_i$ ($mg\ g^{-1}\ min^{1/2}$) is the intra-particle diffusion rate constant.

1) When the adsorption process is controlled by liquid membrane diffusion, there is a linear relationship between t and $In(Q_e - Q_t)$, and the straight line passes through the coordinate origin;

2) When there is a linear relationship between $t^{0.5}$ and $Q_t$ and passes through the the coordinate origin, meaning that the diffusion process of the substance in the particle is the only control step of the adsorption rate;

3) The pseudo-two-level kinetic model includes all the processes of adsorption, such as external liquid membrane diffusion, surface adsorption and particle internal diffusion.

The calculation results of pseudo-first-order and pseudo-second-order kinetics of Cu(II), Cd(II), Pb(II) adsorption by 6O-MWCNTs@$Fe_3O_4$ were shown in the Fig. S4 and Table II. It is seen that $Q_e$ values obtained by the pseudo-second-order are close to those obtained in our experiments. The $R^2$ values of quasi-first order kinetics are 0.993, 0.955, and 0.964 respectively, which are lower than those of pseudo-second-order kinetics (0.999, 0.999, 0.993). These results indicated that the

adsorption kinetics were well described by the pseudo-second-order kinetic model. That is, the adsorption process of metal ions onto adsorbents may be dominated by chemical adsorption process. The fitting for intra-particle diffusion model shows linear relationship between $Q_t$ and $t^{0.5}$ and the regression lines do not pass through the coordinate origin, which indicates that intra-particle diffusion was not the rate-limiting step in the adsorption process (Fig. S3c). The value of $K_1$ is much higher than that of $K_2$, which suggests that the external surface adsorption is the main rate-determining step.

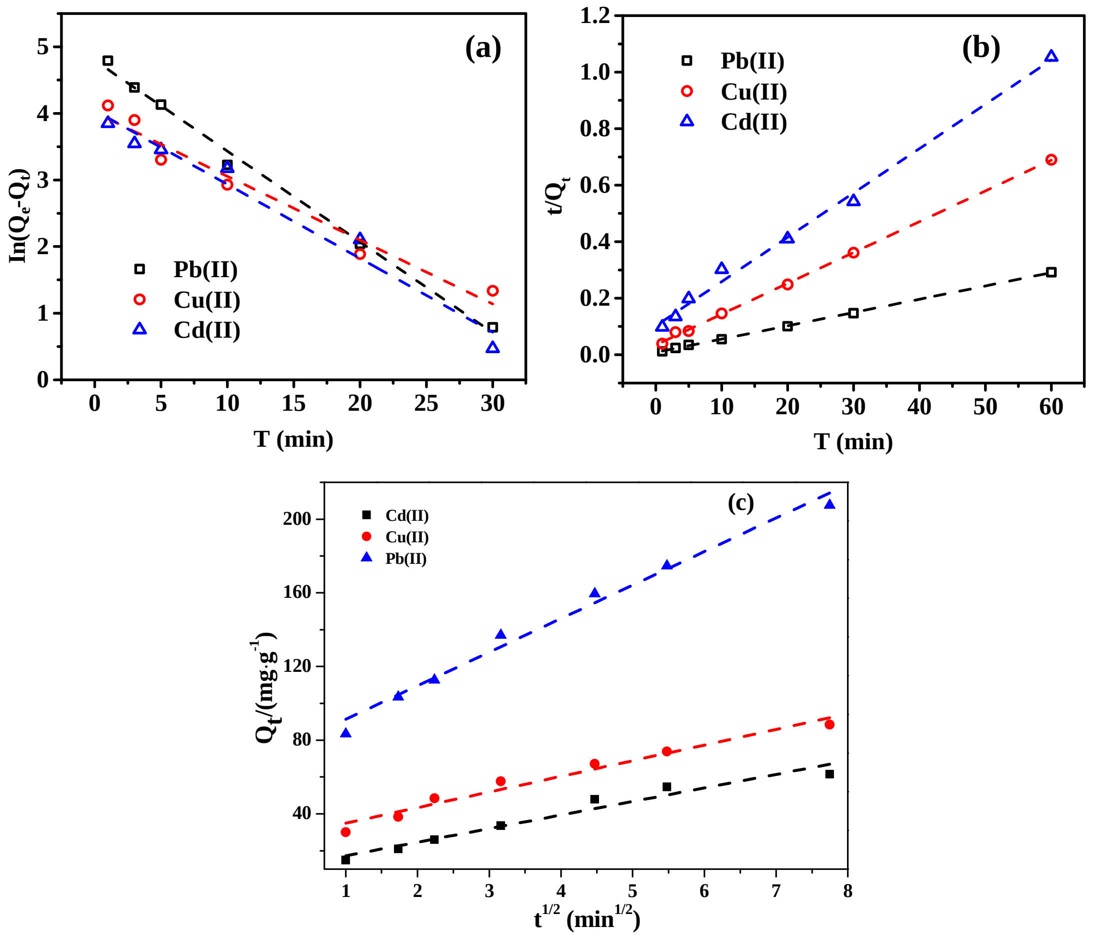


Fig. S3 Pseudo-first-order kinetics model (a), pseudo-second-order kinetics model (b), and internal particle diffusion model (c) of 6O-MWCNTs@$Fe_3O_4$ for Cu(II), Cd(II), Pb(II)

Table I Calculation results of pseudo-first-order and pseudo-second-order kinetics of 6O-MWCNTs@$Fe_3O_4$ for Cu(II), Cd(II), and Pb(II).

| Metal ions | Pseudo-first -order | | | Pseudo-second-order | | | |
|---|---|---|---|---|---|---|---|
| | $K_1$ | $Q_e$ | $R^2$ | $K_2$ | $Q_e$ | $h_0$ | $R^2$ |
| Pb(II) | 0.1362 | 120.89 | 0.993 | 0.0026 | 212.77 | 117.7 | 0.999 |
| Cu(II) | 0.0959 | 55.43 | 0.955 | 0.0034 | 91.74 | 28.62 | 0.999 |
| Cd(II) | 0.1110 | 57.03 | 0.964 | 0.0024 | 63.73 | 9.748 | 0.993 |

Langmuir and Freundlich isotherm adsorption models were used to describe the

adsorption behavior of Cd(II), Cu(II) and Pb(II) on 6O-MWCNTs@$Fe_3O_4$ in the solution.

The Langmuir model is described as follows:

$$\frac{C_e}{Q_e}=\frac{C_e}{Q_m}+\frac{1}{K_L Q_m} \qquad \text{(Eq. 4)}$$

The Freundlich isotherm is expressed as follows:

$$\lg Q_e=\lg K_F+\frac{1}{n}\lg C_e \qquad \text{(Eq. 5)}$$

$Q_e$ (mg/g) is the equilibrium adsorption capacity;

$C_e$ (mg/L) represents the concentration of equilibrium;

$Q_m$ (mg/g) represents the maximum adsorption capacity;

$K_L$ (L/mg) is the Langmuir constant;

$n$ (mg$^{(1-(1/n))}$·L$^{(1/n)}$/g) and $\boldsymbol{K_F}$ (mg$^{(1-(1/n))}$·L$^{(1/n)}$/g) are Freundlich constants and adsorption intensity, respectively.

Adsorption isotherm shows the relationship between adsorption capacity at a constant temperature and the equilibrium concentration of the solution. The fitting curves of Langmuir and Freundlich adsorption isotherms and the relevant parameters were shown in the Fig. S4 and Table II. The results show that the correlation coefficients are fitted much better with Langmuir model ($R^2 > 0.99$) than the Freundlish model ($R^2 > 0.91$), which suggests that all sites of adsorption on the adsorbent have equal affinity to adsorbates and monolayer adsorption process is homogeneous. For the Freundlich isotherm, the value of n ($n > 2$) indicated that Cd(II), Cu(II) and Pb(II) ions are favorably adsorbed onto the surface of 6O-MWCNTs@$Fe_3O_4$. The maximum adsorption capacities of 6O-MWCNTs@$Fe_3O_4$ for Pb(II), Cu(II) and Cd(II) are 215.05, 87.41, 57.18 mg/g, respectively, which is very close to the theoretical calculated value of $Q_m$ (255.17, 90.37, 57.65 mg/g). These fitting calculation results show that the process of 6O-MWCNTs@$Fe_3O_4$ adsorbing metal ions is more in line with the Langmuir isotherm adsorption model.

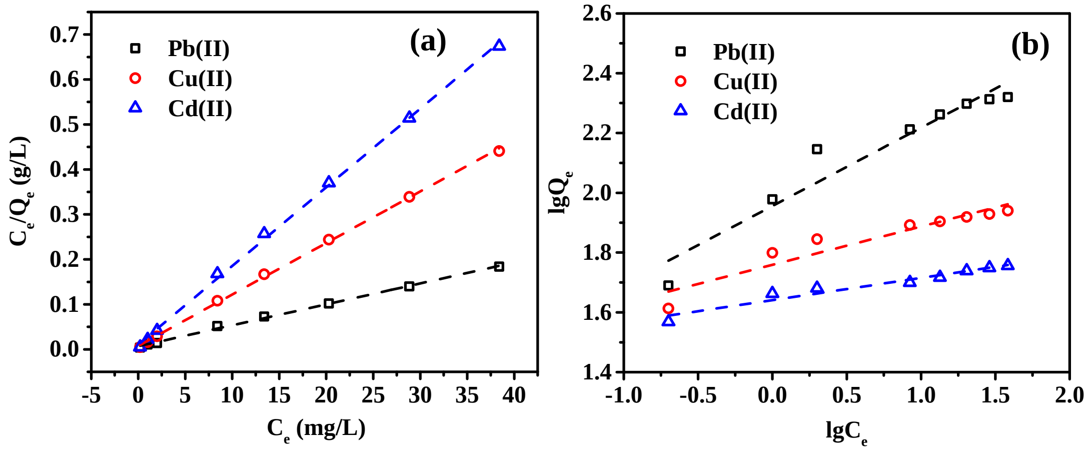


Fig. S4 Langmuir adsorption isotherms (a) and Freundlich adsorption isotherms (b) of 6O-MWCNTs@$Fe_3O_4$ for Cu(II), Cd(II), and Pb(II)

Table II Calculation results of 6O-MWCNTs@$Fe_3O_4$ for Cu(II), Cd(II) and Pb(II) isothermal adsorption model

| Metal ions | Langmuir model | | | Freundlich model | | |
|---|---|---|---|---|---|---|
| | $Q_m$ (mg/g) | $K_L$ (L/mg) | $R^2$ | $n$ | $K_F$ | $R^2$ |
| Pb(II) | 258.17 | 0.123 | 0.997 | 3.819 | 90.33 | 0.919 |
| Cu(II) | 90.37 | 0.562 | 0.999 | 7.823 | 57.43 | 0.886 |
| Cd(II) | 57.65 | 0.854 | 0.998 | 13.48 | 43.75 | 0.938 |
